# Defeating Huanglongbing Pathogen *Candidatus* Liberibacter asiaticus With Indigenous Citrus Endophyte *Bacillus subtilis* L1-21

**DOI:** 10.3389/fpls.2021.789065

**Published:** 2022-01-21

**Authors:** Shahzad Munir, Yongmei Li, Pengbo He, Pengfei He, Pengjie He, Wenyan Cui, Yixin Wu, Xingyu Li, Qi Li, Sixiang Zhang, Yangsu Xiong, Zhanjun Lu, Wenbiao Wang, Kexian Zong, Yongchao Yang, Shaocong Yang, Chan Mu, Heming Wen, Yuehu Wang, Jun Guo, Samantha C. Karunarathna, Yueqiu He

**Affiliations:** ^1^State Key Laboratory for Conservation and Utilization of Bio-Resources in Yunnan, Yunnan Agricultural University, Kunming, China; ^2^Binchuan Institute for Food and Medicine Inspection and Testing, Binchuan, China; ^3^College of Life Sciences, Gannan Normal University, Ganzhou, China; ^4^Institute of Upland Crops, Wenshan Academy of Agricultural Sciences, Wenshan, China; ^5^Institute of Crop Fertilization, Yuxi Academy of Agricultural Sciences, Yuxi, China; ^6^Key Laboratory of Economic Plants and Biotechnology, Kunming Institute of Botany, Chinese Academy of Sciences (CAS), Kunming, China; ^7^Institute of Tropical and Subtropical Cash Crops, Yunnan Academy of Agricultural Sciences, Baoshan, China; ^8^Center for Mountain Futures (CMF), Kunming Institute of Botany, Chinese Academy of Sciences (CAS), Kunming, China

**Keywords:** Citrus, *Bacillus subtilis*, endophyte, pathogen, restructuring, microbiome

## Abstract

Huanglongbing (HLB) has turned into a devastating botanical pandemic of citrus crops, caused by *Candidatus* Liberibacter asiaticus (*C*Las). However, until now the disease has remained incurable with very limited control strategies available. Restoration of the affected microbiomes in the diseased host through the introduction of an indigenous endophyte *Bacillus subtilis* L1-21 isolated from healthy citrus may provide an innovative approach for disease management. A novel half-leaf method was developed *in vitro* to test the efficacy of the endophyte L1-21 against *C*Las. Application of *B. subtilis* L1-21 at 10^4^ colony forming unit (cfu ml^−1^) resulted in a 1,000-fold reduction in the *C*Las copies per gram of leaf midrib (10^7^ to 10^4^) in 4 days. In HLB-affected citrus orchards over a period of 2 years, the *C*Las incidence was reduced to < 3%, and *C*Las copies declined from 10^9^ to 10^4^ g^−1^ of diseased leaf midribs in the endophyte L1-21 treated trees. Reduction in disease incidence may corroborate a direct or an indirect biocontrol effect of the endophytes as red fluorescent protein-labeled *B. subtilis* L1-21 colonized and shared niche (phloem) with *C*Las. This is the first large-scale study for establishing a sustainable HLB control strategy through citrus endophytic microbiome restructuring using an indigenous endophyte.

## Introduction

Huanglongbing (HLB) disease, a major uncontrollable disease of citrus trees resulting in significant yield losses, is caused by one or a combination of the phloem-inhabiting α-proteobacteria *Candidatus* Liberibacter asiaticus (*C*Las), *Ca*. Liberibacter africanus (*C*Laf), and *Ca*. Liberibacter americanus (*C*Lam) (Bové, 2006). The bacterium *C*Las may cause imbalances in host metabolism due to consumption, competition, and depletion of host nutrients (Duan et al., 2009). The main vector of *C*Las is Asian citrus psyllid (ACP), *Diaphorina citri* (Narouei-Khandan et al., 2016), and understanding the mechanisms involved in plant-pathogen interaction is essential for the development of novel HLB management strategies (Albrecht and Bowman, 2008; Fan et al., 2011).

The characteristics of HLB disease symptoms include blotchy mottled and pale yellow leaves, followed by distinct yellow shoots, corky veins, stunting, lopsided fruits with color inversion, and twig dieback (Ajene et al., 2020). Roots are also observed with a dramatic reduction in the fibrous root mass of infected plants, which leads to poorly developed root systems (Johnson et al., 2014; Li et al., 2019). Citrus-infected plants with HLB are not always but confused easily with symptoms of nutrient deficiency and without professional training (Tian et al., 2014) because the pathogen distribution is always highly patchy, as leaves and stems contain most of the bacterial titers (Li et al., 2006). In the beginning, the titer may be the highest in the roots (Johnson et al., 2014; da Rocha et al., 2019). The symptoms are clearer and more apparent in the cooler season compared to warmer months. Shimwela et al. (2019) estimated that the incubation period can be several months to years. It is still unknown that how long the trees have been infected before the appearance of symptoms, but symptomatic citrus trees eventually show disease symptoms (Dala-Paula et al., 2019).

So far, there are no curative methods to control this pathogen; although, scientists have used a few control measures to eliminate or reduce the pathogen growth. Approaches to control HLB include frequent use of antibiotics such as penicillin (Shin et al., 2016; Ascunce et al., 2019; McVay et al., 2019) and oxytetracycline (Blaustein et al., 2018), screening for small molecule inhibitors (Pagliai et al., 2015), combinations of stock and scion (Albrecht et al., 2016), graft-based chemotherapy (Zhang et al., 2012; Yang et al., 2016) and transgenic technology (Hao et al., 2016; Zou et al., 2017), control of psyllid vector (Tomaseto et al., 2019), and, finally, the most extreme measure is chopping off and burning down the diseased trees, which results in polluting environment even more. However, this method may slow down the disease, but with huge losses.

However, biocontrol of plant diseases can be the most promising alternative to continuously failing treatments and endophyte-mediated biocontrol offers consistent results due to intimate association with its host and shared niche with the pathogen where endophyte could fully manifest their antagonistic potential, whereas the non-endophytic biocontrol agents often fail under field conditions due to fierce competition in the rhizosphere and harsh environmental conditions (Ahmed et al., 2020; Blacutt et al., 2020; Trivedi et al., 2020). In biocontrol programs, native endophytes could be used to maintain long-term colonization inside the citrus host and eliminate *C*Las through niche and nutrient competition (Munir et al., 2018b). Biocontrol of plant pathogens has yielded effective results using bacteria under laboratory conditions (Herschkovitz et al., 2005; Andreote et al., 2010; Dematheis et al., 2013) and moderate effects under field conditions (Mattos-Jr et al., 2020), but the potential of indigenous endophytic bacteria as economically useful biocontrol agents against citrus pathogens has not been investigated, especially for HLB control (Munir et al., 2021). This approach may bring a revolution in managing citrus disease caused by phloem-limited α-proteobacteria (Bové, 2006).

Plant diseases and various plant protection measures lead to perturbations of the host microbiome (Irigoyen et al., 2020); it is also evident that *C*Las is associated with changes in microbial population dynamics during the HLB disease progression (Zhang et al., 2013a, 2016; Munir et al., 2019); therefore, it is of immense importance to understand the impact of key endophytic bacteria in the citrus health and defense against HLB. It is suggested that the key inhibitors of *C*Las play an important role in pathogen suppression (Barnett et al., 2019).

In an effort to gain in-depth insight, the aim of this study was to quantify the effects of regular applications of an indigenous endophyte, *Bacillus subtilis* L1-21 isolated from healthy citrus trees (selected based on potential biocontrol activities against several bacterial and fungal pathogens) to diseased trees for *C*Las reduction. This study highlights the promising research findings of using a novel citrus half-leaf method to reduce the *C*Las in the presence of *B. subtilis* L1-21. Most importantly, the evidence was provided from the two field experiments in the presence of endophyte L1-21, where citrus was diseased for more than 5 and 2 years, respectively. Finally, we point out that the red fluorescent protein (RFP)-tagged endophyte L1-21 shared the same niche with *C*Las inside citrus phloem. To the best of our knowledge, this is the first study undertaking a citrus endophyte-mediated management against HLB disease on a large field scale with consistent control.

## Materials and Methods

### Bacterial Strain and Culture Conditions

The endophytic strain *B. subtilis* L1-21, an indigenous endophyte isolated from a healthy citrus host (Munir et al., 2020a) and deposited in the Chinese Culture Collection Bank, Beijing (Accession number: CGMCC15726), was selected for the greenhouse and citrus field experiments (Binchuan, Yunnan Province, China). The endophyte *B. subtilis* L1-21 and its RFP-tagged strain were stored in 40% glycerol (v/v) at −80°C; stock culture was renewed every 4 months on Luria Bertani (LB) agar and cultured for 24–48 h to check the stability of the potential endophyte. A pure culture of endophyte L1-21 was grown until the late log phase in LB broth for 24–48 h at 37°C in a shaking incubator (150 rpm). The colony forming units (cfu ml^−1^) of the endophyte were checked using contrasting dilutions in sterile distilled water with 0.1% Tween 20 and plated on LB agar before foliar spraying on the citrus trees.

### Novel Citrus Half-Leaf Method

We designed a novel citrus half-leaf method to quantify *C*Las copies in leaves prior to and after treatment based on conventional and quantitative PCR (qPCR). Diseased citrus leaves [*Citrus reticulata* (*C. reticulata*)] were collected randomly from 16 years old citrus grove and brought to the laboratory in ice and immediately processed for experiment or stored in 4°C till further use. Firstly, DNA was extracted from one-half of a diseased leaf using the cetyltrimethylammonium bromide (CTAB) method (Araújo et al., 2002; Munir et al., 2019) and conventional PCR analysis, using the primer set Cal-R/Cal-F (Jagoueix et al., 1996), was followed by nested PCR analysis, using the primer set CG03F/CG05R (Zhou et al., 2007), for confirmation of “*Ca*. Liberibacter asiaticus.” Primers used in this study are given in Supplementary Table S1. Once confirmed as positive for *C*Las, the other half of the citrus leaf (4–5 cm) was suspended with 10^4^-10^6^ cfu ml^−1^ of endophytes in a 5-ml Eppendorf tube and the different antibiotics, i.e., penicillin (100 μg μl^−1^) dissolved in distilled water, spinosad (100 μg μl^−1^) dissolved in hexane solution, and shenqimycin (100 μg μl^−1^) dissolved in distilled water as chemical control (positive). LB broth and sterilized distilled water were used as uninfected control (negative). The Eppendorf tubes were kept for 96 h at room temperature, followed by extraction of DNA from the leaf midribs. The *C*Las 16S ribosomal RNA (16S rRNA) gene was amplified as discussed previously (Munir et al., 2019). The weight of each half leaf was recorded prior to the experiment. Each treatment comprised three replicate Eppendorf tubes that each contained six diseased citrus leaves midribs. The experiment was repeated five times. Treatment effects on *C*Las were tested by loading the amplified product on 1% agarose gel. In another experiment testing for *C*Las and endophytes abundance, diseased leaves were placed in Petri dishes (180 mm) and different treatments were performed. DNA was extracted from one-half of a diseased leaf using the CTAB method (Araújo et al., 2002) and amplified with the same primers as described previously. Once the leaf was confirmed as infected with *C*Las, the other half of the leaf was kept in a 180-mm Petri dish with 10^4^ and 10^6^ cfu ml^−1^ or penicillin (100 μg μl^−1^) dissolved in distilled water. LB and distilled water were used as controls. The Petri dishes were kept at room temperature for an interval of 1 day for collected leaves to be checked for pathogen concentration and endophytes for 4–5 days; then, DNA was extracted from the midribs and present endophytes were isolated (Araújo et al., 2002; Munir et al., 2018b). The weight of each half leaf was recorded prior to the experiment. Each treatment comprised three replicates of 12 diseased citrus leaves (six random top and bottom half leaves, each) and the experiment was repeated five times. The presence of the pathogen was confirmed using standard qPCR analysis and density (copies g^−1^) was calculated based on a standard curve generated by cloning 382 bp of a specific DNA fragment located in the ribosomal protein (*rplJ*) (Munir et al., 2020a). The PCR reaction was performed in a 25-μl reaction mixture containing 1 × PCR buffer (SYBR Green Master Mix; Bio-Rad, United States), 0.8 μl of each primer (CQULA04R/CQULA04F), and the appropriate amount of DNA template in an RT-PCR system (StepOne Real-Time PCR System, Applied Biosystems, United States) as reported previously (Wang et al., 2006). Changes in *C*Las copies were tested from 0 to 4 days, based on CT values of pathogen copies g^−1^ of the citrus leaf that were calculated using the standard curve generated previously; changes in endophyte density were recorded daily as log cfu g^−1^ of citrus leaf midrib.

### Phloem Colonization of *Bacillus subtilis* L1-21 RFP

For confirmation of phloem colonization of the endophyte, the RFP-tagged strain of *B. subtilis* L1-21 was generated using genomic DNA by amplifying the *mKate2* coding sequence with ribosome-binding site. *Bam* H1 and *Hin*d III enzymes were used for product digestion and ligated with the plasmid pYC127 as discussed previously (Chai et al., 2008). The resulting plasmid designated as PY69 was used to make overexpression RFP strain of *B. subtilis* L1-21. A strain expressing red fluorescent reporter gene (*mKate2*) was grown until late log phase in LB medium with 10 μg μl^−1^ chloramphenicol in shaking incubator at 37°C. Citrus plants (2 years old) in the greenhouse were foliar sprayed with RFP-tagged endophytic strain L1-21 and its colonization was visualized by confocal laser scanning microscope (CLSM) after 2 days. Citrus leaves were detached and gently washed for surface sterilization as performed previously to remove unattached cells from the citrus leaves (Munir et al., 2021). Leaves midribs were separated and put on the specimen holder by adding optimal cutting temperature (OCT) compound (Sakura, Europe). Specimen holder containing citrus leaves midribs were sliced briefly on the microtome-cryostat (Leica Biosystems, United States). Slice midribs were examined using CLSM (Olympus, FV10-ASW). The emission of RFP was measured at 405 to 635 nm after excitation at 559 nm, while citrus plant autofluorescence was measured after excitation at 405 and 635 nm for blue and green fluorescence, respectively. Images were captured using an automatic picture program FV1000 Viewer. The experiment was repeated three times, each time with three replicates.

### Study Site and Sample Processing From HLB-Affected Citrus Groves

The experimental field site was located at Binchuan, Dali City, Yunnan Province, China (100.5754° E, 25.8272° N), with an average annual temperature of 33–35°C and total annual rainfall of about 1,000 mm. Foliar symptoms in one citrus grove (*C. reticulata*), 16 years old, indicated > 90% *C*Las disease incidence, and the second citrus grove (*C. reticulata*), 4 years old, which was located 2 km away from the first citrus grove, exhibited 100% *C*Las infection as confirmed through conventional PCR and qPCR before the start of experiments. Two field trials were conducted from 2017 to 2019: in one citrus grove, 162 trees (Supplementary Figure S1) were treated with contrasting treatments and in the second citrus grove, 93 of 525 trees were selected (three replicates of 31 trees) for *C*Las titer test. Prior to the start of each trial, a field survey was carried out to visually determine individual tree HLB severity and detected the *C*Las pathogen using *C*Las-specific primers in PCR and qPCR analyses. The soil treatments at the citrus groves comprised fertilizer 1 (rapeseed cape with *Bacillus amyloliquefaciens* Y2, as a root growth promoting agent); fertilizer 2 (rapeseed cape without *Bacillus amyloliquefaciens* Y2); and no rapeseed cape as a control (F_0_). Antibiotic penicillin (100 μg μl^−1^) dissolved in distilled water was applied one time through trunk injection at the start of the experiment as shown in Supplementary Table S2. In addition, we applied 5 kg of fertilizer to each tree around a water drip of about 20 cm deep. The antibiotics and endophytes used in this study are given in Supplementary Table S2.

### Field Endophyte Treatment Application

The endophyte application was carried in the field experiments. Two injection ports per tree were made by drilling 30 mm into the xylem tissue using a 7.14-cm drill bit, located at the opposite sides of the trunk at ~20 cm above the union bud. Antibiotic and endophyte (*B. subtilis* L1-21) treatments were injected from a pressurized bottle into each port using a micro drip infusioner at the recommended pressure (<50 psi). The first injection was done with penicillin (0.1%) for 4 days on 54 trees, with 27 trees as a control. The second injection was done 2 weeks after the antibiotic treatment with the endophyte (10^6^ cfu ml^−1^) on 54 trees. We also applied 10^6^ cfu ml^−1^ of the endophyte or penicillin as a monthly foliar spray on 27 trees for each treatment, until all leaves were wet, in the early morning or late evening. All the experiments were arranged as three replicates of three trees in a randomized complete block design.

### Isolation of Endophytes From Citrus Trees

*Candidatus* Liberibacter asiaticus causing HLB disease negatively affected the indigenous endophytic microbes. Therefore, we estimated the density of endophytic bacteria from three replicates of six citrus tree leaves that were sampled from the trees and transported to the laboratory in a cooler with ice. Endophytes were isolated from three replicates of treatment samples collected on the first week of each month in 2017–2018 as isolated previously (Araújo et al., 2002).

### *Candidatus* Liberibacter asiaticus DNA Extraction and PCR Amplification

Leaf samples from all the treatments were collected monthly from the citrus groves to quantify *C*Las titer. Leaves were treated as above before DNA extraction. Methods for DNA extraction, PCR, and qPCR were those described previously.

### Statistical Analysis

Endophytic bacteria populations were calculated, based on average logarithm (base 10) cfu values, and analyzed using the GraphPad Prism version 8 (San Diego, California, USA). Pathogen copies g^−1^ of diseased citrus leaf material were calculated using a standard curve value (Munir et al., 2020a). Treatment effects were tested using the ANOVA in the SPSS Statistics version 22.0 (IBM Corporation, Armonk, New York, USA) and treatment means were compared using Duncan's multiple range test at *p* < 0.05.

## Results

### *Bacillus subtilis* L1-21 Suppression of *C*Las in the Laboratory

**Figure 1:**
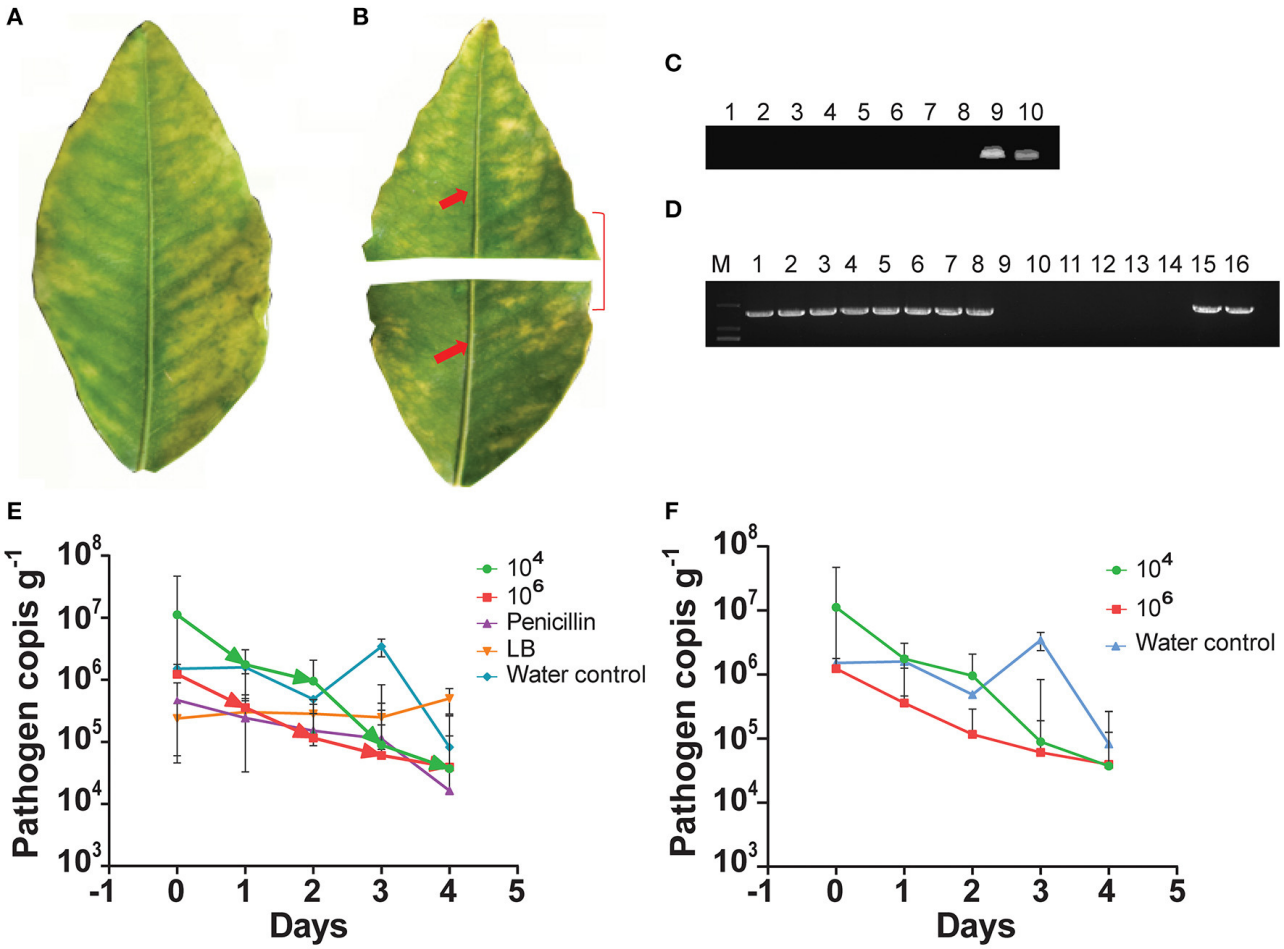
Citrus half-leaf method for reduction of *Candidatus* Liberibacter asiaticus (*C*Las) in the diseased citrus leaves from 0 to 4 days. Sketch of citrus leaf used in novel citrus half-leaf experiment. **(A)** Whole citrus leaves showing Huanglongbing (HLB) symptoms; **(B)** Leaf was parted into two parts; the first part was used to detect the *C*Las pathogen using conventional and quantitative PCR (qPCR); after confirmation, the second part was used in the experiment for *Bacillus subtilis* (*B. subtilis*) L1-21 treatment. Red arrows indicate leaf midribs used for extraction of DNA for conventional and qPCR; **(C)** Conventional PCR and diseased citrus leaves were treated with endophytes (10^3^-10^6^ cfu ml^−1^) and antibiotics penicillin (100 μg μl^−1^ and 500 μg μl^−1^), shenqinmycin (100 μg μl^−1^), and spinosad (100 μg μl^−1^); lane 9, water control; lane 10, positive control; **(D)** Conventional PCR, before application; Lane 1, citrus leaf sample treated with penicillin; Lane 2, leaf samples treated with spinosad; Lane 3, citrus leaves treated with shenqinmycin; Lane 4, sample in Luria Broth (LB) medium treated with endophytes without shaking; Lanes 5 and 6, samples in LB medium and water, respectively, on shaker; Lanes 7 and 15, diseased citrus leaf in water only; Lanes 8 and 16, positive control; after application: Lane 9, indicated reduction of *C*Las pathogen with different treatments including penicillin; Lane 10, spinosad; Lane 11, shenqinmycin; Lane 12, endophyte treatment in LB medium without shaking; Lane 13, endophyte treatment in LB medium with shaking; Lane 14, samples in water with shaking; **(E)** qPCR, pathogen copies per gram of leaves midrib using 10^4^ and 10^6^ colony forming unit (cfu) ml^−1^ of endophyte treatment one time. Pathogen copies g^−1^ was calculated based on the standard curve of recombinant plasmid pUC18-382-HLB generated through qPCR. Penicillin was used as a positive control. LB medium and water were used as negative control treatments. Three biological replicates were used for each treatment and each replicate consisted of 12 diseased leaves (including 6 top and bottom half leaves each). The experiment was repeated five times; and **(F)** Pathogen copies g^−1^ of leaves using 10^4^ and 10^6^ cfu ml^−1^ of endophyte and water treatment one time. The values are means ± SD of five replicated experiments.

We used the indigenous endophyte *B. subtilis* L1-21 against *C*Las in the leaf midrib by means of citrus half-leaf method (Figures 1A,B) maintained under room temperature in Eppendorf tube. *C*Las copies in leaf material were reduced by 5 days after treatment with the endophyte at 10^4^ and 10^6^ cfu ml^−1^ (Figure 1C); similarly, the antibiotics used in this experiment also reduced *C*Las copies (Figure 1D). Subsequent dilutions of the endophyte validated the results, confirming that *C*Las copies inside the citrus leaf midrib were reduced by a single application of the endophyte. Since the leaves midribs were cut and put on shaking in water, we suggested that pathogen ooze out of the midribs and no positive band was observed.

To further confirm the efficacy of the endophyte against *C*Las, we used the half-leaf method to test pathogen copies in one half of the leaf present in Petri dish treated with a single application of the *B. subtilis* L1-21 (10^4^ and 10^6^ cfu ml^−1^) or penicillin antibiotic (as a positive/chemical control) and LB broth and water (as negative/uninfected controls). The presence of the pathogen was confirmed using standard qPCR analysis and density (copies g^−1^) was calculated based on a standard curve (Munir et al., 2020a). CLas pathogen copies in the leaf midribs treated with the endophyte L1-21 (10^4^ and 10^6^ cfu ml^−1^), penicillin, LB, and water were 1.12 × 10^7^, 1.23 × 10^6^, 4.71 × 10^5^, 2.38 × 10^5^, and 1.51 × 10^6^, respectively (Figure 1E; Supplementary Table S3). Application of the endophyte reduced *C*Las copies in the leaf midribs 1,000-fold (1.12 × 10^7^ to 3.72 × 10^4^) and 100-fold (1.23 × 10^6^ to 3.93 × 10^4^) by 4 days after a single treatment of 10^6^cfu ml^−1^ and 10^4^ cfu ml^−1^ (Figure 1F; Supplementary Table S3) of the endophyte, respectively. Application of penicillin reduced *C*Las in diseased citrus leaves 10-fold (4.71 × 10^5^ to 1.64 × 10^4^) and there was no effect of LB or water controls by 4 days after treatment. These results clearly indicated that application of the endophytes reduced CLas pathogen in diseased citrus leaves. The half-leaf method was also employed to check the pathogen reduction in individual leaves using top and bottom parts to confirm validation of results and we showed that *C*Las could be reduced to more than 90% after 3–4 days (Supplementary Table S4).

### *Bacillus subtilis* L1-21-Derived Indigenous Endophytes Inside Citrus Leaves

**Figure 2:**
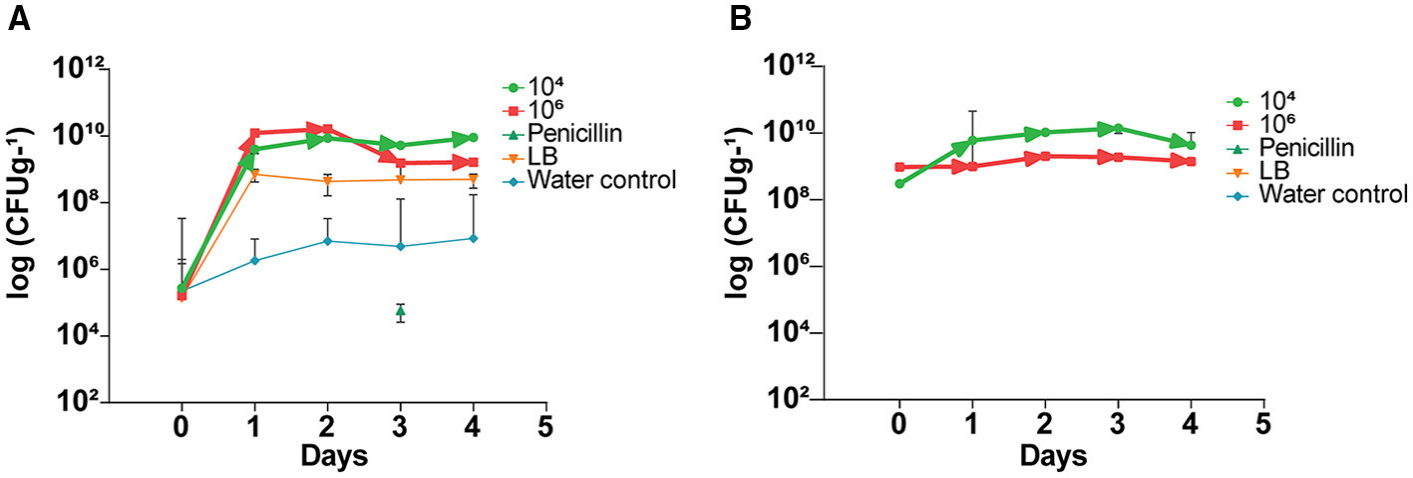
Elevation of indigenous endophytes derived after application of *B. subtilis* L1-21 in citrus half-leaf method. **(A)** Native endophytes density in the diseased citrus leaves after application of endophytes and penicillin and negative control. **(B)** Recovery of treated endophytes from the diseased citrus leaves during different time intervals with different treatments. The populations of endophytic bacteria were calculated based on the average logarithm (base 10) of bacteria recovered from the plant leaves. The detection limit (1 × 10^2^ cfu leaves^−1^) was treated as log 0 for mean calculation. The log cfu values were analyzed with the GraphPad Prism version 8 (San Diego, California, USA). The values are means ± SD of five replicated experiments.

Prior to application of the endophyte, indigenous citrus endophyte density in the citrus leaves was 10^5^ cfu g^−1^; following a single treatment with endophytic strain L1-21, the density of other endophytes present inside leaves increased to 10^9^ cfu g^−1^ (Figure 2A). Treated endophyte L1-21, which displayed pinkish color on LB media, was recovered from the diseased citrus leaves during different time intervals with different treatments mentioned (Figure 2B). The application of penicillin initially decreased leaf midrib microbe density, but this effect subsided with time as the endophyte density returned to pretreatment levels. The endophyte density was unaffected by the LB and water treatments.

### *Bacillus subtilis* L1-21 Colonization in Citrus Phloem

**Figure 3:**
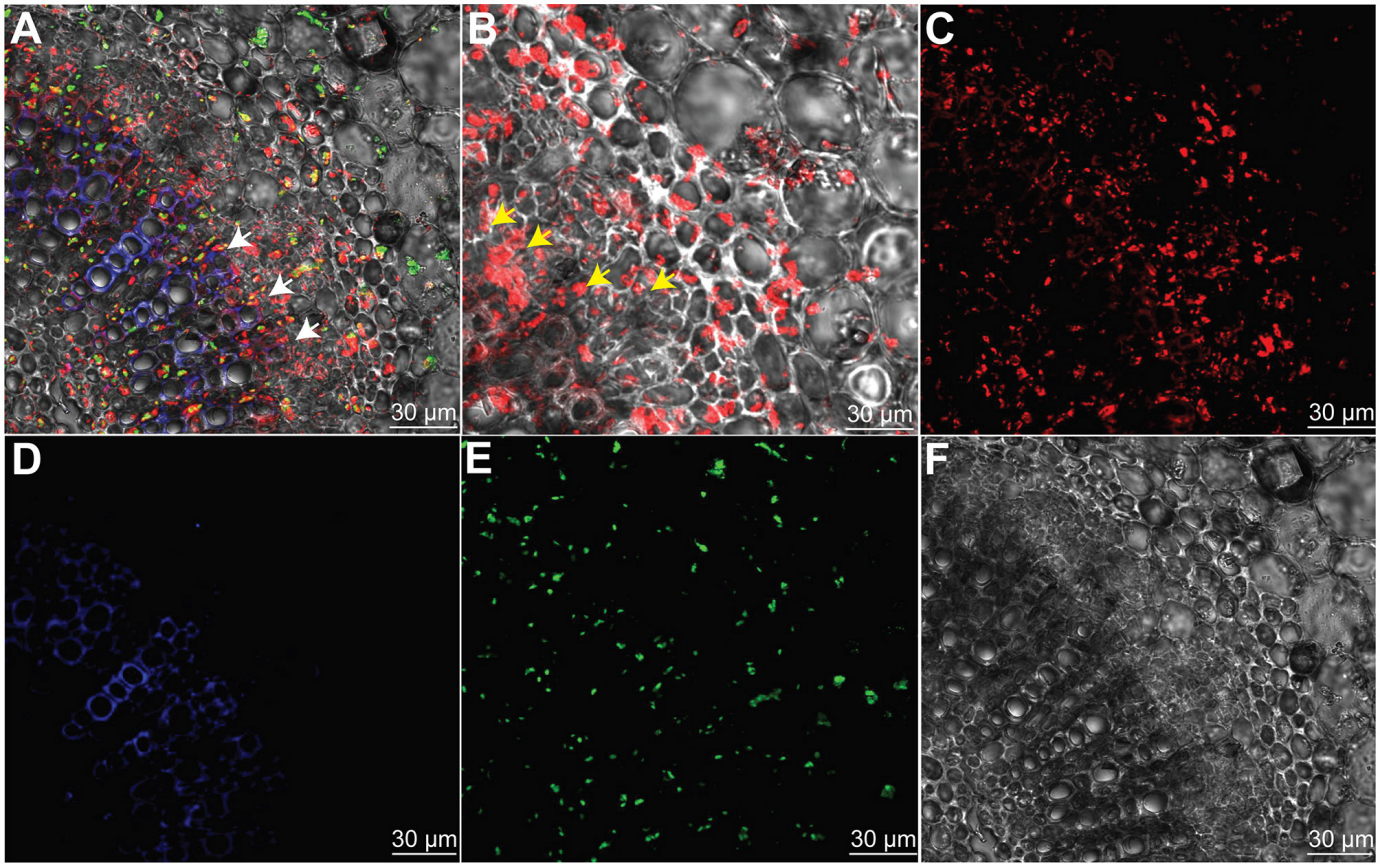
*Bacillus subtilis* L1-21 colonization in the phloem of citrus leaves midribs. **(A–E)** Indigenous citrus endophyte expressing *mKate2* was grown until the late log phase and foliar sprayed on citrus plants in the greenhouse. Confocal laser scanning microscope was carried for visualization of *B. subtilis* L1-21 red fluorescent protein (*RFP*) in phloem; **(A)** Transverse section of merged citrus leaves midrib displaying red fluorescence (phloem); green autofluorescence (chlorophyll); blue fluorescence depicted the xylem; **(B)** Enlarge part from panel A observed for details colonization of endophyte inside citrus leaves phloem; **(C)** Individual RFP of *B. subtilis* L1-21; **(D)** Xylem with blue autofluorescence; **(E)** Green autofluorescence generated by chlorophyll; and **(F)** Dark field showing citrus phloem. The scale represents the 30 μM. The white and yellow arrows represent the localization of RFP-tagged *B. subtilis* L1-21 inside phloem.

To prove the hypothesis that the indigenous endophyte *B. subtilis* L1-21 colonization inside citrus midribs phloem helps in excluding the *C*Las, which resides in citrus phloem, an RFP-tagged L1-21 strain was constructed that can express *mKate2* genes. After observation with CLSM, we found that RFP-tagged L1-21 successfully colonizes the citrus midribs (Figure 3). Two days after inoculation, we observed that a lot of cells are present inside the phloem. These results indicated that the endophyte not only colonizes the citrus phloem completely but also reduces the pathogen load present in the citrus phloem as proved above.

### Efficacy of Short-Term Field Applications of *Bacillus subtilis* L1-21 Against *C*Las

**Figure 4:**
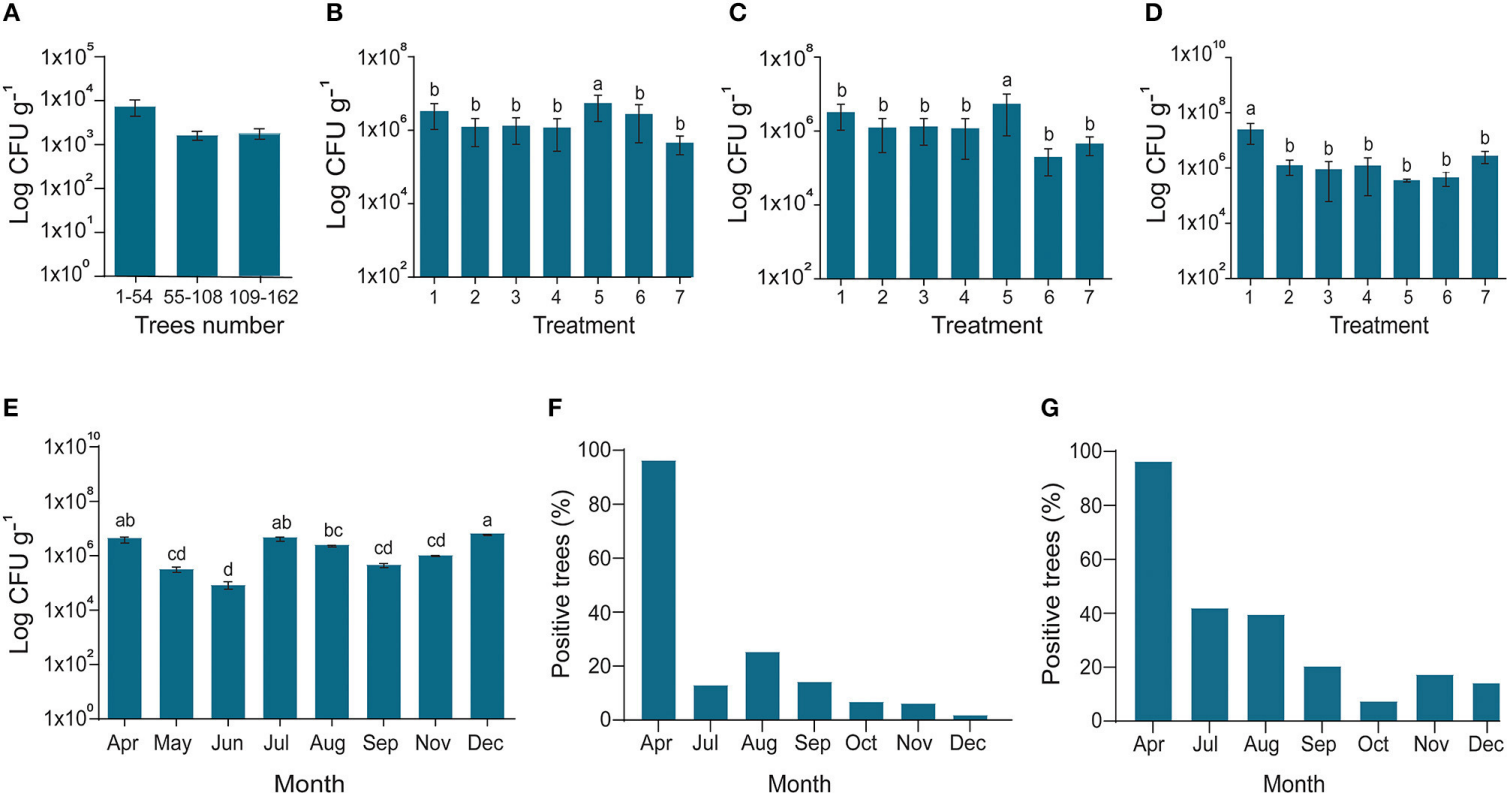
Endophytes density and reduction of *C*Las pathogen. **(A)** In March 2017, the diseased citrus trees were checked for possible assessment endophyte before starting experiment; **(B)** In April 2017, all the trees treated with fertilizer 1 with *Bacillus amyloliquefaciens* Y2; the X-axis represents endophytes and penicillin injection through the trunk (1); endophyte injection (2); Penicillin spray (3); Endophyte spray (4); Penicillin injection (5); Control CK1 and CK2 (6,7); **(C)** Fertilizer 2 with *B. amyloliquefaciens* Y2; CK2 and CK3 (6,7); **(D)** No organic fertilizer (F_0_) was used as control; (6,7) indigenous endophytes showing increase in the number of endophytes due to dispersal of endophytes in all the trees even control samples; **(E)** Endophytes population during different sampling times along with; and **(F)** Successive reduction of *C*Las pathogen inside disease trees using conventional PCR and **(G)** Nested PCR. The populations of endophytic bacteria were calculated based on the average logarithm (base 10) of bacteria recovered from the plant leaves. The log cfu values were analyzed with the GraphPad Prism version 8 (San Diego, California, USA). The values are means ± SD with statistically significant difference among different treatments with different letters (*p* ≤ 0.05).

In 2017–2018, the efficacy of potential endophyte *B. subtilis* L1-21 was tested against *C*Las in two diseased citrus groves (>90% and 100% HLB prevalence, respectively) containing 16- and 4-year-old citrus trees, respectively. We treated the *C*Las-infected mandarin groves with contrasting applications of the endophyte, antibiotic, and biofertilizers to determine the most effective management strategy (Supplementary Figure S1). HLB-affected citrus grove was completely recovered after 6–7 months of successful treatment with the indigenous endophyte (Supplementary Figures S1B,C). Antibiotic and biofertilizers were applied once at the start of the experiment and endophytes were applied monthly, following leaf sampling to check densities of *C*Las and the endophyte. Prior to the start of the experiment, all the citrus trees were tested for the total number of endophytes present inside each tree (Figure 4A). *Bacillus subtilis* L1-21 applied to the citrus trees spreads to neighboring, untreated trees (Supplementary Figure S2); therefore, the endophytic density of all the citrus trees were presented on monthly basis. The density of *C*Las and endophytes was measured monthly in leaf samples collected from each of the 162 citrus trees using conventional and nested PCR techniques (Figures 4B–D). The health of leaves was improved following treatment with the endophyte for 1 year, where they changed from yellow to green, and we found negative effects of *C*Las on the endophytic microbiomes of the citrus tree. Citrus trees were monitored monthly to assess visual disease symptoms and following 1 year of monthly applications of *B. subtilis* L1-21, *C*Las density in the trees reduced significantly. In the first quarter of the experiment, the number of diseased trees reduced to <100, where trees that had been characterized by yellow and mottled leaves began to develop more robust shoots and leaves. The leaf density of citrus endophytes was initially reduced by the application of antibiotic but later recovered to more than 10^7^ cfu g^−1^. By the second quarter of the experiment, the number of diseased trees reduced to 69, in which the endophyte density was similarly increased; by the final quarter, the number of diseased citrus trees reduced to three, and trees in the grove finally yielded fruit suitable for the commercial market. Thus, we confirmed that the endophyte *B. subtilis* L1-21 was the most effective control agent for the management of HLB and a reliable yet cheap option for citrus growers worldwide. Strengthening citrus microbe density using an endophyte with diverse antagonistic activities through trunk injection and foliar spray application may elevate microbe density in diseased citrus trees to levels where *C*Las may more easily be controlled (Figure 4E). Previously, we found the number of the endophyte types was lower in diseased citrus trees, indicating that greater density of indigenous citrus endophytes may contribute to the control of *C*Las in diseased trees (Munir et al., 2019).

**Figure 5:**
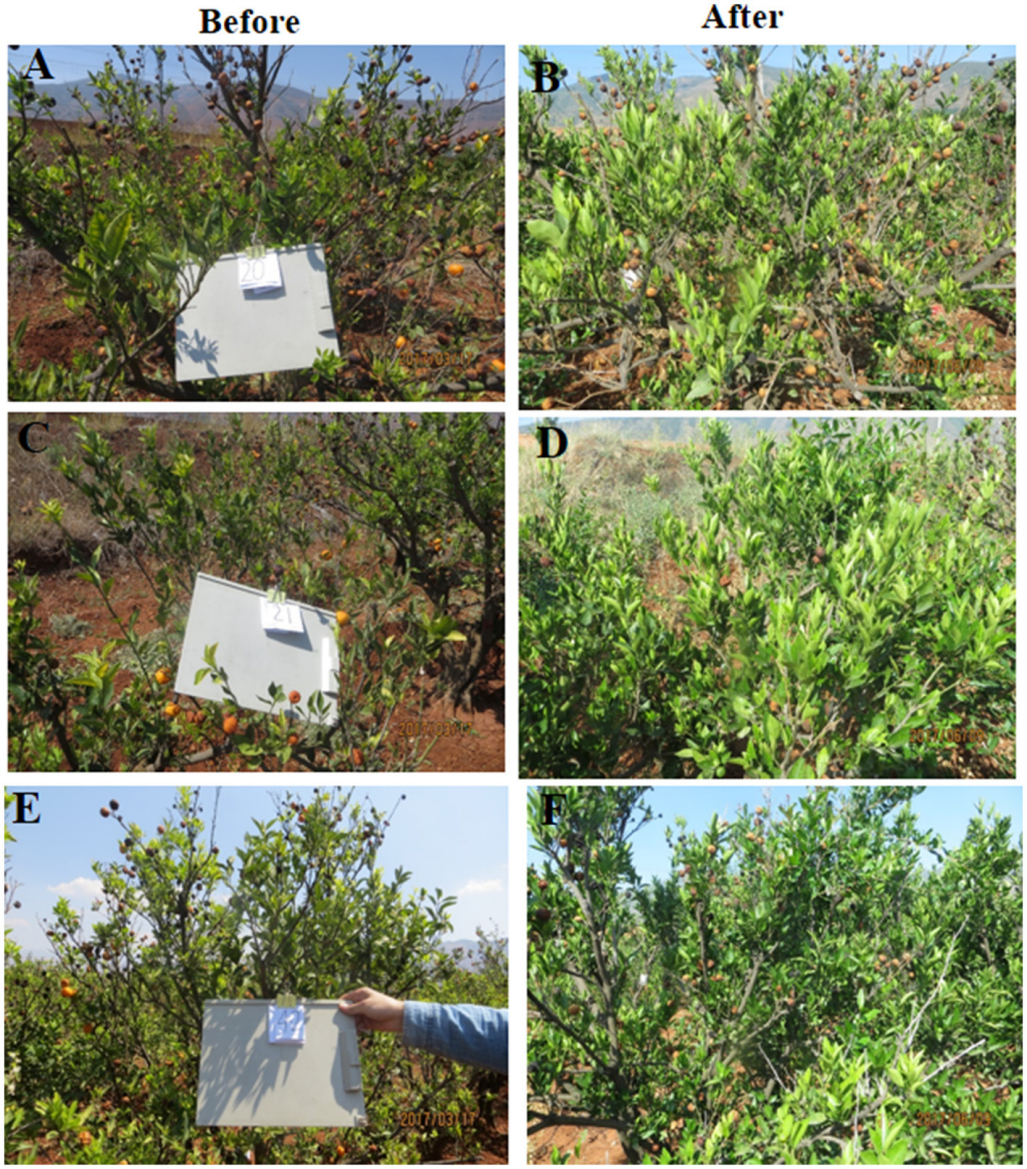
Huanglongbing affected diseased citrus groves before and after application of endophyte L1-21 in Binchuan county, Yunnan Province, China. The citrus groves were checked for *C*Las pathogen detection before the start of the experiment to make sure that all the citrus trees are affected with HLB. **(A,C,E)** In March 2017, the citrus grove was affected with HLB and **(B,D,F)** In June 2017, trees displayed more robust growth with green leaves after *Bacillus subtilis* L1-21 application.

**Figure 6:**
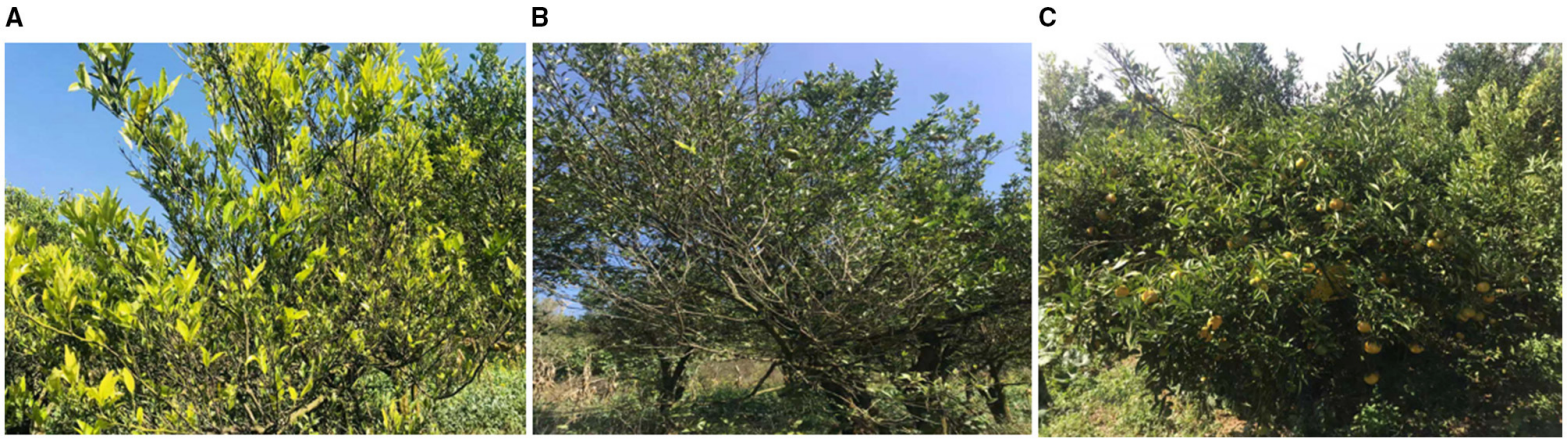
Management of HLB in diseased citrus fields using endophyte *Bacillus subtilis* L1-21: In 2018, an experiment was performed to check the reduction of diseased severity in a citrus grove in Genma county, Yunnan Province, China. Trees showed diseased symptoms to a healthy state after 7–8 months regular treatment with indigenous citrus endophyte L1-21. **(A)** April 2018; **(B)** June 2018; and **(C)** November 2018 (Genma county, Yunnan Province, China).

The copies of *C*Las in leaf samples were reduced following 6 months of monthly treatments with *B. subtilis* L1-21 (Figures 4F,G), confirming our hypothesis that indigenous citrus endophytes may reduce disease incidence in the field by >95%. After a year of monthly applications of the endophyte, population of *B. subtilis* L1-21 in citrus leaf midribs increased from 10^3^ to around 10^9^ cfu g^−1^ in most trees, showing that indigenous endophytes may represent a novel management strategy for the control of *C*Las in citrus plants (Figure 5). We have found similar effects of this endophyte on *C*Las in 2 other citrus groves in the study region and one each in the Genma county of Yunnan Province (Figure 6).

### Effects of Long-Term Field Applications of *Bacillus subtilis* L1-21 on *C*Las

**Figure 7:**
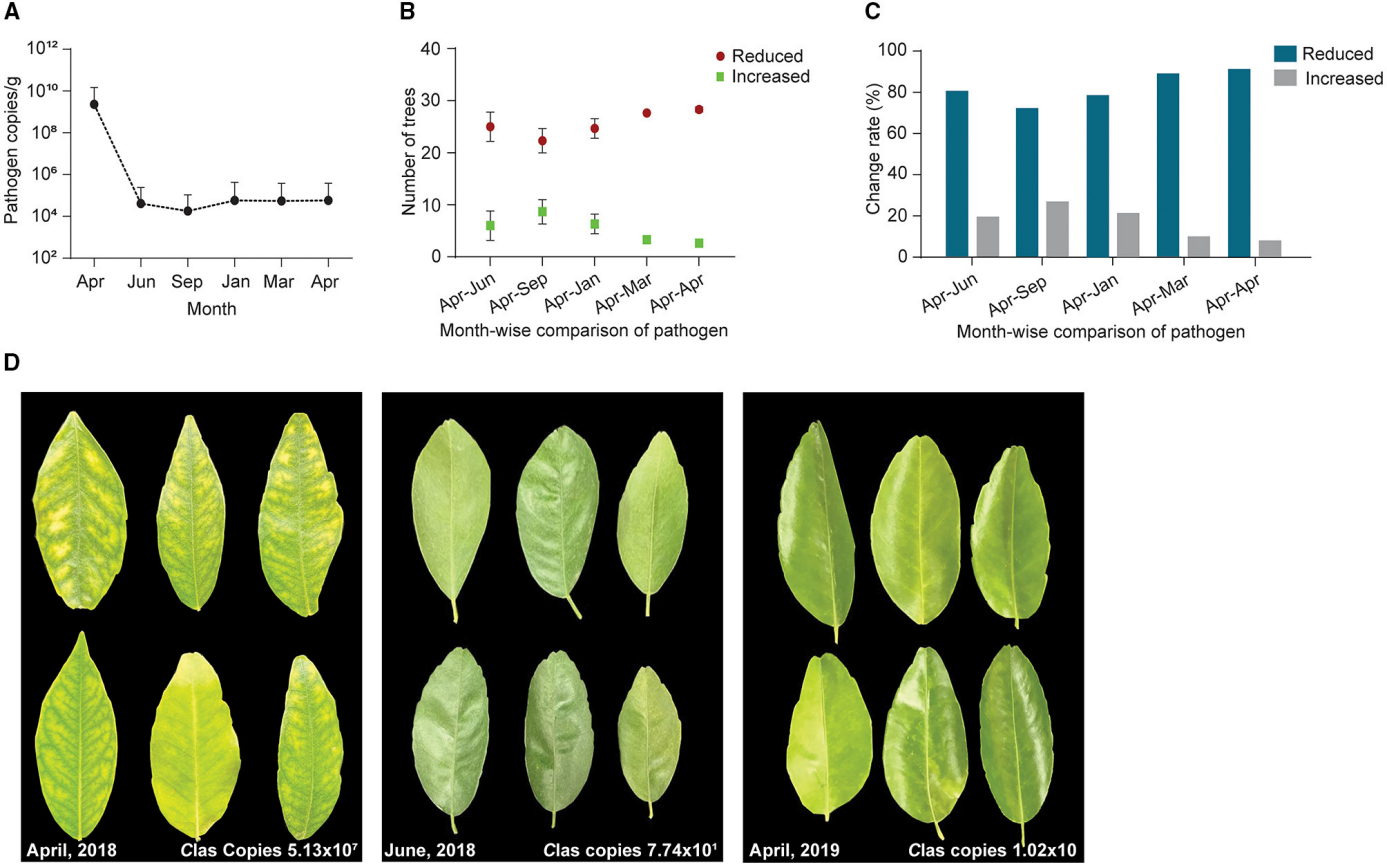
A total of 93 citrus diseased sampled trees were treated with endophytes and *C*Las pathogen was monitored throughout the study period from April 2018 to April 2019. **(A)** CT values obtained through qPCR were changed to pathogen copies per gram of citrus leaf using a standard curve generated from pUC18-382 recombinant plasmid; **(B)** Number of citrus trees displayed a reduction of *C*Las pathogen during different time intervals, April to September 2018 and January, March, and April 2019. Each repeat contains 31 citrus trees; **(C)** Percentage reduction of pathogen across month-wise indicating reduction of the pathogen from time to time; and **(D)** Citrus leaves showing yellow symptoms before application of endophytes (April 2018) to more greenish leaves with the different time intervals decreasing pathogen copies 10^7^ to 10 *C*Las/gram citrus leaf midribs.

To confirm the results from the 1-year field experiments, we regularly treated diseased citrus groves, comprising 525 trees, with *B. subtilis* L1-21 in 2018–2019; these regular applications reduced copies of *C*Las in the citrus trees so that the number of diseased trees was reduced. Finally, we selected 93 among the 525 trees, which represented three replicates of 31 trees and found that after 1 year, regular applications of the endophyte had reduced the *C*Las from 10^9^ to 10^4^ g^−1^ of leaf midrib (Figure 7A) (99% control; Table 1). *C*Las copies were very high in April 2018 but decreased within 3 months (Figure 7B). In April 2019, we found 16 of the 93 diseased trees contained 100 copies of the pathogen g^−1^ of leaf material, while 39 diseased trees contained < 100 copies, representing a 91.39% reduction in *C*Las (Figure 7C) and there was a decrease in the number of yellow leaves and shoots and an increase in the growth of new shoots (Figure 7D). Our findings indicated that *C*Las density remained constant in around 25% of the citrus trees during the endophyte treatment and was undetected in 16 of the diseased citrus trees in April 2019. Thus, we suggested that *C*Las was successfully eliminated from diseased citrus trees by *B. subtilis* L1-21.

**Table 1:** Reduction of *Candidatus* Liberibacter asiaticus (*C*Las) pathogen copies during different time intervals 2018–2019.

***C*** **Las copies in diseased citrus grove**					
**Month**	**I**	**II**	**III**	**Mean**	**Reduce from April**
April, 2018	8.34 ×10^8^	4.98 ×10^9^	3.68 ×10^5^	1.94 ×10^9^	-
Jun, 2018	4.26 ×10^3^	2.24 ×10^4^	3.86 ×10^4^	2.18 ×10^4^	99.9989%
Sept, 2018	8.91 ×10^3^	3.31 ×10^4^	4.59 ×10^3^	1.55 ×10^4^	99.9992%
Jan, 2019	2.62 ×10^4^	1.44 ×10^5^	1.57 ×10^3^	5.74 ×10^4^	99.9970%
March, 2019	2.64 ×10^4^	1.29 ×10^5^	9.76 ×10^3^	5.51 ×10^4^	99.9971%
April, 2019	2.09 ×10^4^	1.52 ×10^5^	1.72 ×10^3^	5.81 ×10^4^	99.9970%

*Repeat I, II, and III: Each consists of 31 citrus trees. The CLas reduction (pathogen copies g^−1^) was tested during 1-year period. Each of these values displayed in Table 1 represents the average pathogen copies of 31 trees. Each tree was randomly sampled for 12–15 leaves*.

## Discussion

In order to demonstrate the specific role of endophytes to manage the alarmingly devastating disease of citrus, we isolated and identified indigenous endophytes from symptomatic, asymptomatic, and healthy leaves from different locations of citrus growing regions in China (Munir et al., 2020a). We tested the efficacy of *B. subtilis* L1-21, which showed marked pink colonies differed among all the isolated endophytes, used as a potential endophyte isolated from citrus trees in the experimental fields. The novel half-leaf method advanced the understanding of pathogen exclusion within the midrib. The endophyte application positively affected other microbes in citrus leaves, indicating native bacterial density was unaffected. It is likely that healthy and asymptomatic citrus trees harbor disease suppressive endophytic bacteria that may secrete antimicrobial compounds in the host phloem and, perhaps, contribute to protection against *C*Las. We found that there was no effect on *C*Las copies in diseased citrus leaves of treatment with LB and water as controls, indicating that reduction of the pathogen within the leaf was due to application of *B. subtilis* L1-21. Previously, citrus endophytes, such as *Methylobacterium* and *Sphingomonas*, were also found to be involved in the protection of plants against various devastating pathogens (Innerebner et al., 2011; Ardanov et al., 2012).

The pathogen reduction is associated with competition for the nutrients among pathogen and endophytes and substances released by endophytes that could have possible interference with the pathogen quorum sensing signaling (Miller and Bassler, 2001; Piewngam et al., 2018). Endophytic strain *B. subtilis* L1-21 was labeled with RFP that can express *mKate2* gene. In light of our finding, CLSM visualization clearly showed successful colonization in the phloem of citrus leaves, which may open more doors for future research to study the mechanisms involved in the interaction of the endophyte with non-culturable *C*Las. The endophyte colonization in a similar niche as *C*Las might have employed direct inhibition of pathogen by producing antibiotics or lipopeptides, bacteriocin proteins (Kamada et al., 2013). On the other hand, core microbiomes present in the citrus host may be regulated by these endophytes, creating the possibility of pathogen elimination, most probably through induced systemic resistance (ISR) (Pertot et al., 2013; Zhao et al., 2018).

Currently, *C*Las is being treated with antibiotics (oxytetracycline hydrochloride, penicillin G potassium, ampicillin, and others) in the greenhouse and field experiments, but these are phytotoxic and persist as residues inside fruits (Zhang et al., 2014; Munir et al., 2018a). Proper use of antibiotics at the right time, adequate dosage, and application techniques can avoid problems related to the residue and phytotoxicity (McVay et al., 2019). Dissemination of antibiotics in the agriculture system is a potential threat to human health, agricultural productivity, natural ecosystem functioning, development of resistance in the pathogen, and negative effects on native microbiota (Williams-Nguyen et al., 2016), which urge the implementation of environmental-friendly alternatives for disease control. The use of antibiotics in citrus groves is common practice, which has been shown to drastically reduce the bacterial community richness and diversity (Zhang et al., 2013b); however, studies focusing on antibiotic effect solely on an endophytic community are scarce. Other common management practices such as glyphosate applications between the trees likely affect the endophytic microbial community over time. Our field experiment demonstrated successful management of *C*Las in HLB-diseased citrus with regular applications of *B. subtilis* L1-21. Pathogen copies in the diseased trees were reduced to <10 g^−1^ of leaf midrib and growth of green leaves and shoots was increased, including in previously moribund trees; similarly, marketable fruit yields also improved. However, the underlying mechanisms of endophytes on reductions in *C*Las copies and disease development remain unclear. These results indicated disease suppressive activities, such as quorum sensing attenuation by pathogen diffusible antibacterial compounds, niche and nutrient competition, synthesis of plant growth promoting hormones, siderophores or other bioactive compounds, and induction of plant defense, may have occurred with field applications of the endophyte, which limited in planta transmission of the pathogen, but warrants detailed study (Munir et al., 2021). Further, we assume that reduction of the pathogen may be related to increasing within plant endophyte density because the previous study has documented that *C*Las present inside citrus leaves regulated HLB symptoms positively and the leaf microbiome negatively (Blaustein et al., 2017; Munir et al., 2019). Pathogen exclusion effects were related to the increased density of *B. subtilis* L1-21 within the diseased trees. To determine *in planta* endophyte interactions with *C*Las, we first quantified their pretreatment densities in the diseased trees. Following treatment with *B. subtilis* L1-21, the endophyte densities in the citrus trees increased and the density of pathogen reduced. Notably, high copies of *C*Las remained present in untreated diseased trees. Thus, this study confirmed that the population dynamics of native citrus bacteria affect *C*Las titers (Sagaram et al., 2009; Zhang et al., 2016). It has been suggested that the endophyte application may also inhibit pathogen indirectly through activation of plant defense or recruitment of other endophytes in the citrus that causes the *C*Las reduction. We found significant genes and pathways associated with the elimination of *C*Las. Some pathways were effectively regulated in the diseased host, but not in healthy trees after endophytes treatment confirming different roles of endophytes in the host (data not shown).

Timing and effective dosage of application are much important to use biocontrol in field conditions (Brilli et al., 2019). Desired results can be achieved using this endophyte in the field during any time of the year in citrus fields. Later, we found that this endophyte can colonize for a longer duration inside citrus plants and maximum abundance was present even after 3 months, which makes it economically feasible for farmers. Using antibiotics to manage HLB disease could raise major concerns with respect to the uptake and distribution of antibiotics in citrus plants (Hijaz et al., 2020). Antibiotics such as penicillin and oxytetracycline showed uneven distribution in different parts of citrus plants and activity against the disease was also not stable (Al-Rimawi et al., 2019). In addition, endophyte-based biocontrol products will cost 100 dollars/hectares during 3 months of application and citrus growers and overall industry could minimize the use of high costing antibiotic applications (Sundin and Wang, 2018).

**Figure 8:**
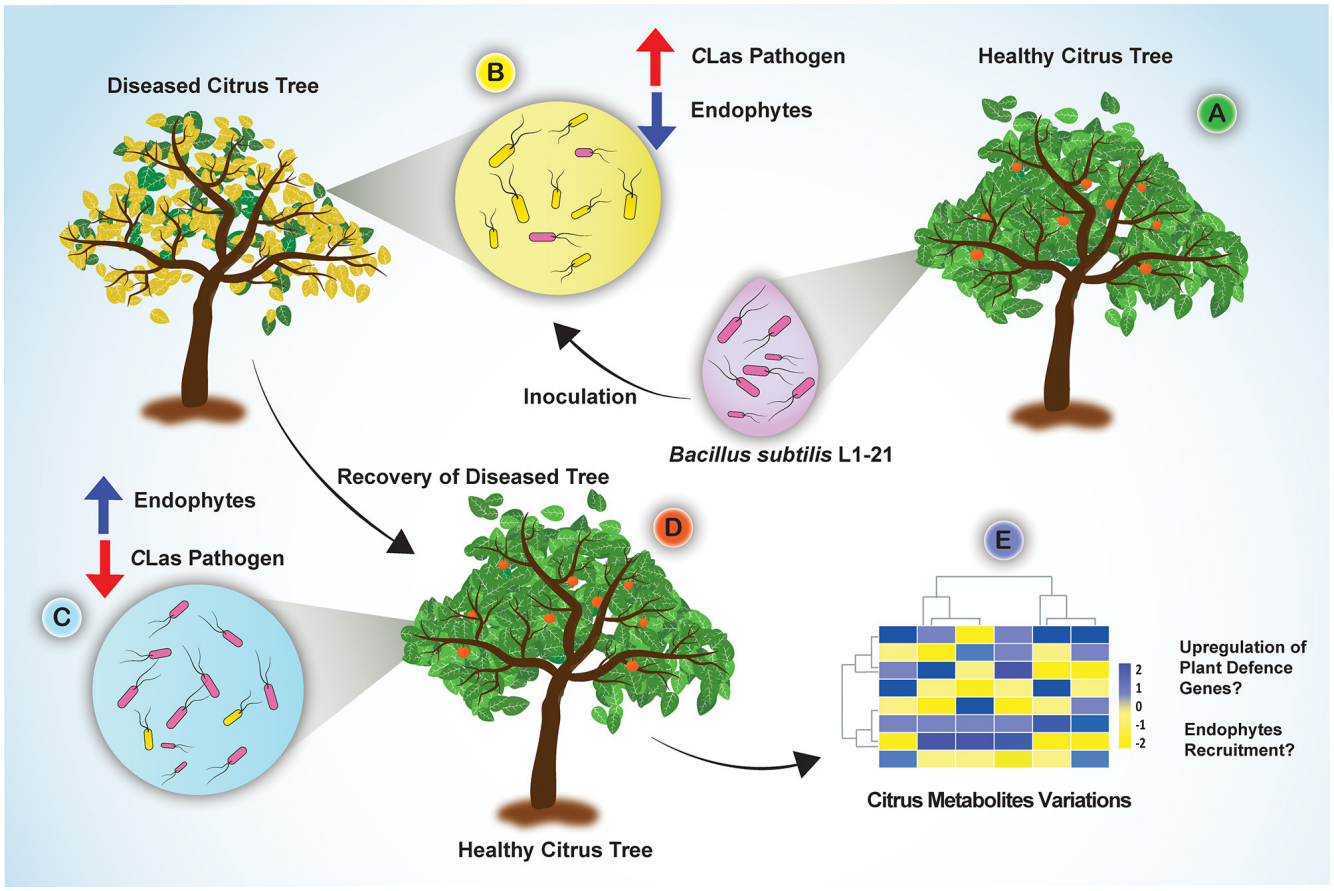
Concluding sketch illustrating the different events taking place in the diseased citrus fields through restructuring bacterial endophytes for management of HLB disease. **(A)** Indigenous endophyte *B. subtilis* L1-21, displaying marked pinkish colonies, was isolated from healthy citrus plants; **(B)** Diseased citrus trees with more pathogen and less endophytes were inoculated with the endophyte *B. subtilis* L1-21; **(C)** Endophyte L1-21 shifted the bacterial endophytic community and excluded/restricted the pathogen colonization in diseased citrus trees from two different regions by regular application for 1 year; **(D)** The recovered tree showing more robust leaves and fruits; **(E)** Significant citrus metabolites were upregulated in the diseased citrus host after application of potential endophyte *B. subtilis* L1-21 (Munir et al., 2020b).

*Candidatus* Liberibacter asiaticus density in citrus trees is driven by an inversely proportional density of endophytes. Potential endophyte *B. subtilis* L1-21 for the management of citrus HLB using novel half-leaf method *in vitro* demonstrated that treatment of diseased citrus leaves reduced pathogen density in leaf midribs by 1,000-fold. In addition, phloem localization of endophyte L1-21 has also been confirmed in citrus revealed the possible role of pathogen exclusion. In light of our finding, this endophyte can efficiently colonize the phloem of citrus leaves, which may open more doors for future research to study the mechanisms involved in the interaction of endophytes with vascular pathogens. Based on the results, how the potential endophyte leads to a restructuring of the bacterial endophytic microbiome in diseased citrus groves is given in Figure 8. We suggested that indigenous endophyte application reduces the pathogen inside diseased citrus canopy from >90 to <3% within a year to a level that will bring benefit to the farmer in the future. Although the suppressive mechanism of endophyte against pathogen requires further exploration, however, we gained valuable insights about multiple metabolites variations in HLB-affected citrus trees, which mounted a defense against pathogen posttreatment with endophyte (Munir et al., 2020b). This study is the first initiative to highlight the restructuring of the citrus bacterial endophytic microbiomes by indigenous endophytes to mitigate the devastating disease HLB in economically important citrus crops.

## Data Availability Statement

The original contributions presented in the study are included in the article/Supplementary Material, further inquiries can be directed to the corresponding author.

## Author Contributions

SM, YL, PengfH, PengbH, PengjH, WC, YiW, YuW, JG, SK, and XL performed the laboratory experiments and data analyses. SM, SZ, ZL, YX, WW, KZ, ZL, YY, QL, SY, CM, HW, and YH performed field experiments and collected samples, and supervised the study. SM and YH drafted the manuscript. All authors contributed to the article and approved the submitted version.

## Funding

This study was financially supported by the National Natural Science Foundation of China (32050410307), the Central Government Fund for Local Science and Technology Development (202107AA110007), the China Postdoctoral Science Foundation (No. 2020M683664XB), and the Yunnan First Level Research Fund for Post-Doctorate Researchers (202103).

## Conflict of Interest

The authors declare that the research was conducted in the absence of any commercial or financial relationships that could be construed as a potential conflict of interest.

## Publisher's Note

All claims expressed in this article are solely those of the authors and do not necessarily represent those of their affiliated organizations, or those of the publisher, the editors and the reviewers. Any product that may be evaluated in this article, or claim that may be made by its manufacturer, is not guaranteed or endorsed by the publisher.
