# Corrigendum: Defeating Huanglongbing Pathogen *Candidatus* Liberibacter asiaticus With Indigenous Citrus Endophyte *Bacillus subtilis* L1-21

**DOI:** 10.3389/fpls.2022.884890

**Published:** 2022-03-29

**Authors:** Shahzad Munir, Yongmei Li, Pengbo He, Pengfei He, Pengjie He, Wenyan Cui, Yixin Wu, Xingyu Li, Qi Li, Sixiang Zhang, Yangsu Xiong, Zhanjun Lu, Wenbiao Wang, Kexian Zong, Yongchao Yang, Shaocong Yang, Chan Mu, Heming Wen, Yuehu Wang, Jun Guo, Samantha C. Karunarathna, Yueqiu He

**Affiliations:** ^1^State Key Laboratory for Conservation and Utilization of Bio-Resources in Yunnan, Yunnan Agricultural University, Kunming, China; ^2^Binchuan Institute for Food and Medicine Inspection and Testing, Binchuan, China; ^3^College of Life Sciences, Gannan Normal University, Ganzhou, China; ^4^Institute of Upland Crops, Wenshan Academy of Agricultural Sciences, Wenshan, China; ^5^Institute of Crop Fertilization, Yuxi Academy of Agricultural Sciences, Yuxi, China; ^6^Key Laboratory of Economic Plants and Biotechnology, Kunming Institute of Botany, Chinese Academy of Sciences (CAS), Kunming, China; ^7^Institute of Tropical and Subtropical Cash Crops, Yunnan Academy of Agricultural Sciences, Baoshan, China; ^8^Center for Mountain Futures (CMF), Kunming Institute of Botany, Chinese Academy of Sciences (CAS), Kunming, China

**Keywords:** Citrus, *Bacillus subtilis*, endophyte, pathogen, restructuring, microbiome

In the original article, there was a mistake in **Supplementary Datasheet 1 Figure S3** that was an extended part of **Figure 5** as published. **Figure S3** should be deleted from the supplementary section and **Supplementary Figures S3A–D** be removed from “*Efficacy of Short-Term Field Applications of Bacillus subtilis L1-21 Against CLas*” from the **Results** section in the main manuscript paragraph 2. The correction has been made to the **Supplementary Datasheet**.

In the original article, there was a mistake in Figure 4B. The statistic was mentioned incorrectly due to some typo mistakes during writing as published. Figure 4 should be replaced with a modified version. The corrected Figure 4 appears below.

**Figure 4:**
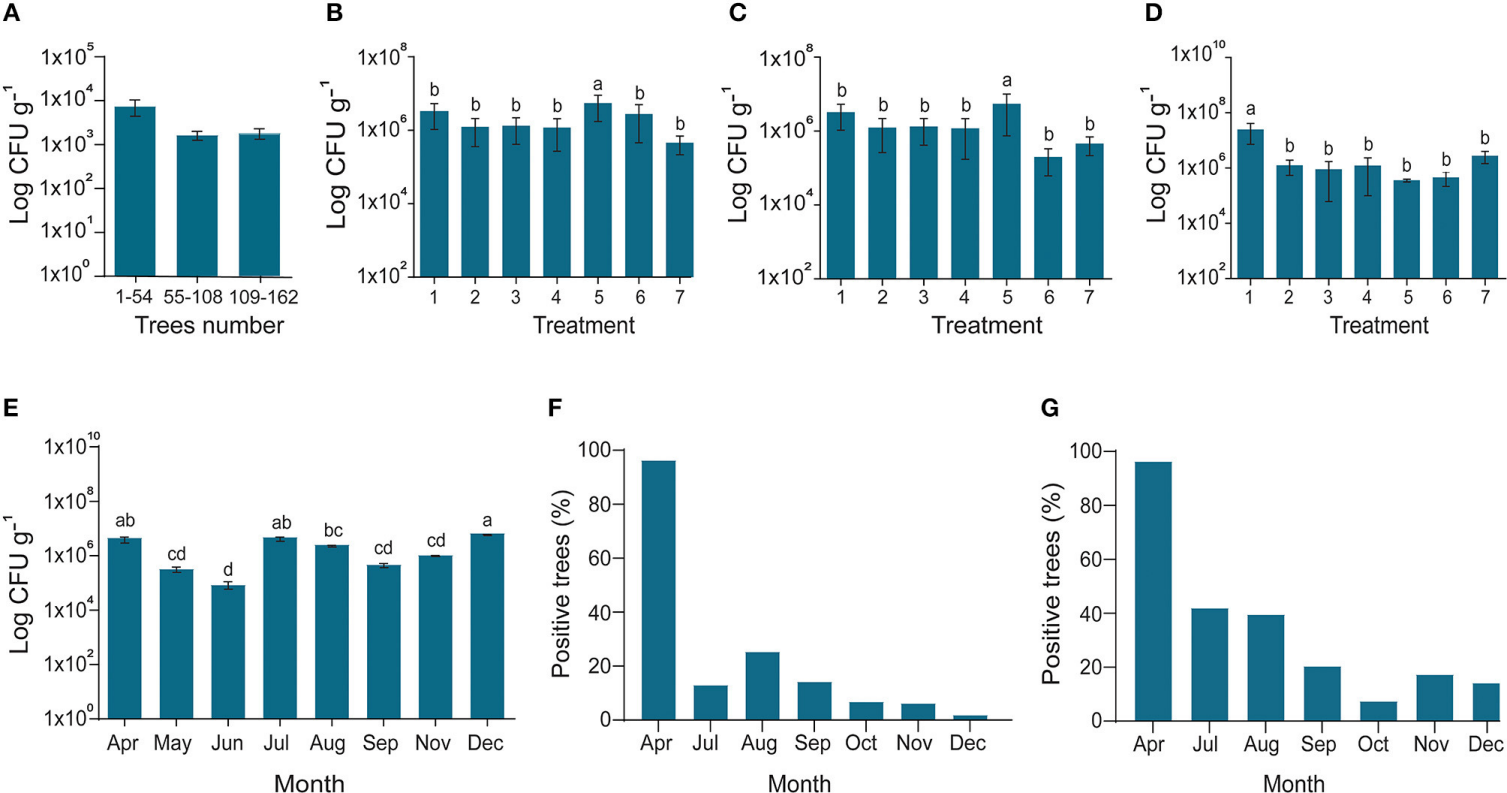
Endophytes density and reduction of *C*Las pathogen. **(A)** In March 2017, the diseased citrus trees were checked for possible assessment endophyte before starting experiment; **(B)** In April 2017, all the trees treated with fertilizer 1 with *Bacillus amyloliquefaciens* Y2; the X-axis represents endophytes and penicillin injection through the trunk (1); endophyte injection (2); Penicillin spray (3); Endophyte spray (4); Penicillin injection (5); Control CK1 and CK2 (6,7); **(C)** Fertilizer 2 with *B. amyloliquefaciens* Y2; CK2 and CK3 (6,7); **(D)** No organic fertilizer (F_0_) was used as control; (6,7) indigenous endophytes showing increase in the number of endophytes due to dispersal of endophytes in all the trees even control samples; **(E)** Endophytes population during different sampling times along with; and **(F)** Successive reduction of *C*Las pathogen inside disease trees using conventional PCR and **(G)** Nested PCR. The populations of endophytic bacteria were calculated based on the average logarithm (base 10) of bacteria recovered from the plant leaves. The log cfu values were analyzed with the GraphPad Prism version 8 (San Diego, California, USA). The values are means ± SD with statistically significant difference among different treatments with different letters (*p* ≤ 0.05).

In the original article, there was an error in the sentence “Each treatment comprised three replicate Eppendorf tubes that each contained six diseased citrus leaves.”

The correction has been made to **Materials and Methods**, “*Novel Citrus Half-Leaf Method*,” paragraph 1:

The new sentence should now read “Each treatment comprised three replicate Eppendorf tubes that each contained six diseased citrus leaves midribs.”

In the original article, there was an error in the sentence “amplifying the *mKate2* coding sequence with ribosome-binding site sequence.”

The correction has been made to **Materials and Methods**, “*Phloem Colonization of Bacillus subtilis L1-21 RFP*,” paragraph 1:

The new sentence should now read “amplifying the *mKate2* coding sequence with ribosome-binding site.”

In the original article, there was an error in “10 μg/μl^−1^.”

The correction has been made to **Materials and Methods**, “*Phloem Colonization of Bacillus subtilis L1-21 RFP*,” paragraph 1:

It should now read as “10 μg μl^−1^.”

In the original article, there was an error in the sentence “*C*Las infection as confirmed through conventional and qPCR before the start of experiments.”

The correction has been made to **Materials and Methods**, “*Study Site and Sample Processing From HLB-Affected Citrus Groves*,” paragraph 1:

The new sentence should now read “*C*Las infection as confirmed through conventional PCR and qPCR before the start of experiments.”

In the original article, there was an error in the sentence “and quantify *C*Las titers using *C*Las-specific primers in PCR and qPCR analyses.”

The correction has been made to **Materials and Methods**, “*Study Site and Sample Processing From HLB-Affected Citrus Groves*,” paragraph 1:

The new sentence should now read “and detected the *C*Las pathogen using *C*Las-specific primers in PCR and qPCR analyses.”

In the original article, there was an error in the sentence “Subsequent dilutions of the endophyte validated the results, confirming that *C*Las copies inside the citrus leaf midrib were reduced by a single application of the endophyte.”

The correction has been made to **Results**, “*Bacillus subtilis L1-21 Suppression of CLas in the Laboratory*,” paragraph 1:

The new sentence should now read as “Subsequent dilutions of the endophyte validated the results, confirming that *C*Las copies inside the citrus leaf midrib were reduced by a single application of the endophyte. Since the leaves midribs were cut and put on shaking in water, we suggested that pathogen ooze out of the midribs and no positive band was observed.”

In the original article, there was an error in the sentence “(100% HLB prevalence).”

The correction has been made to **Results**, “*Efficacy of Short-Term Field Applications of Bacillus subtilis L1-21 Against CLas*,” paragraph 1:

The sentence should now read “(>90% and 100% HLB prevalence, respectively).”

In the original article, there was an error in “**Supplementary Datasheet 1** as **Supplementary Figure S4**” is mentioned.

The correction has been made to **Discussion**, paragraph 3:

There is no Supplementary Figure S4 and this mention has been deleted.

In the original article, there was an error in the sentence “In addition, endophyte-based biocontrol products will cost 10 dollars/hectares.”

The correction has been made to **Discussion**, paragraph 3:

The sentence should now read “In addition, endophyte-based biocontrol products will cost 100 dollars/hectare.”

The authors apologize for this error and state that this does not change the scientific conclusions of the article in any way. The original article has been updated.

## Publisher's Note

All claims expressed in this article are solely those of the authors and do not necessarily represent those of their affiliated organizations, or those of the publisher, the editors and the reviewers. Any product that may be evaluated in this article, or claim that may be made by its manufacturer, is not guaranteed or endorsed by the publisher.

