# Supplementary material for: Defeating Huanglongbing Pathogen Candidatus Liberibacter asiaticus With Indigenous Citrus Endophyte Bacillus subtilis L1-21
Source: Front Plant Sci. 2022 Jan 21;12:789065. doi: 10.3389/fpls.2021.789065 (PMC8813962; doi:10.3389/fpls.2021.789065)
Supplement: Supplementary file 1 [file Data_Sheet_1.docx]

**Supplementary Materials for**

**Defeating Huanglongbing Pathogen *Candidatus* Liberibacter Asiaticus with Indigenous Citrus Endophyte *Bacillus Subtilis* L1-21**

**Authors:** Shahzad Munir,^1^ Yongmei Li,^1^ Pengbo He,^1^ Pengfei He,^1^ Pengjie He,^1^ Wenyan Cui,^1^ Yixin Wu,^1^ Xingyu Li,^1^ Qi Li,^1^ Sixiang Zhang,^2^ Yangsu Xiong,^2^ Zhanjun Lu,^3^ Wenbiao Wang,^2^ Kexian Zong,^2^, Yongchao Yang,^4^ Shaocong Yang,^5^ Chan Mu,^5^ Heming Wen,^4^ Yuehu Wang,^6^ Jun Guo,^7^ Samantha C. Karunarathna,^8^ & Yueqiu He^1,^**^†^**

**Affiliations:**

^1^State Key Laboratory for Conservation and Utilization of Bio-resources in Yunnan, Yunnan Agricultural University, Kunming 650201, Yunnan, China.

^2^Binchuan Institute for Food and Medicine Inspection and Testing, Binchuan 671600, Yunnan, China.

^3^College of Life Sciences, Gannan Normal University, Ganzhou, Jiangxi 360702, China.

^4^Institute of Upland Crops, Wenshan Academy of Agricultural Sciences, Wenshan 663000, Yunnan, China.

^5^ Institute of Crop Fertilization, Yuxi Academy of Agricultural Sciences, Yuxi 653100, Yunnan, China.

^6^ Key Laboratory of Economic Plants and Biotechnology, Kunming Institute of Botany,

Chinese Academy of Sciences, Kunming 650201, Yunnan, China.

^7^Institute of Tropical and Subtropical Cash Crops, Yunnan Academy of Agricultural Sciences, Baoshan 678600, Yunnan, China.

^8^Center for Mountain Futures (CMF), Kunming Institute of Botany, Chinese Academy of Sciences, Kunming 650201, China

**†Corresponding author email:**

**Yueqiu He**

Email: [ynfh2007@163.com](mailto:ynfh2007@163.com)

**Running title: Huanglongbing control with bacterial citrusBiome**

S. Munir, Y. Li and P. He contributed equally to this work.

**Supplementary tables and figures**

**Table S1.** List of all the primers used in this study.

| Gene | Primer | Primer sequence（5’—3’） | Product size (bp) | Reference |
| --- | --- | --- | --- | --- |
| *C*las confirmation | | | | |
| 16S rDNA | CAL-F | GCGCGTATGCAATACGAGCGGCA | 1160 | Jagoueix *et al*., 1996 |
|  | CAL-R | GCCTCGCGACTTCGCAACCCAT |  |  |
| Β-operon gene | CG03F | RGGGAAAGATTTTATTGGAG | 703 | This study |
|  | CG05R | GAAAATAYCATCTCTGATATCGT |  |  |
| rplA/rplJ gene | LAA2 | TATAAAGGTTGACCTTTCGAGTTT | 703 | Hocquellet *et al*., 1999 |
|  | LAJ5 | ACAAAAGCAGAAATAGCACGAACAA |  |  |
| qPCR | | | | |
| rplJ-10 | CQULA03F | CAAGGAAAGAGCGTAGAA | 382 | Wang et al., 2006 |
|  | CQULA03R | CCTCAAGATCGGGTAAAG |  |  |
| rplJ-12 | CQULA04F | TGGAGGTGTAAAAGTTGCCAAA | 87 | Wang et al., 2006 |
|  | CQULA04R | CCAACGAAAAGATCAGATATTCCTCTA |  |  |

**Table S2.** Tree numbers of diseased citrus grove (162 trees) used in the first field treated with *Bacillus subtilis* L1-21.

| **Treatment** | **Organic fertilizer** | | | | | | |
| --- | --- | --- | --- | --- | --- | --- | --- |
|  | **F1** with Y2 | | **F2** without Y2 | | **F0** (no organic fertilizer） | | |
|  | Penicillin | No | Penicillin | No | Penicillin | No | |
| **Endophytic injection** | 1-9* | 10-18 | 55-63 | 64-72 | 109-117 | 118-126 | |
| **Spray** | 19-27 | 28-36 (L1-21) | 73-81 | 82-90  (L1-21) | 127-135 | 136-144  (L1-21) | |
| **Injection** | 37-45 | 46-54(CK1) | 91-99 | 100-108(CK2) | 145-153 | 154-162(CK3) | |
|  | | | | | | | |
| The soil treatments at the citrus groves comprised fertilizer 1 (rape seed cape with *Bacillus amyloliquefaciens* Y2 (10^6^CFU/g), as a root-growth promoting agent); fertilizer 2 (rape seed cape without *Bacillus amyloliquefaciens* Y2); and, no rape seed cape as a control (F_0_). *46-54 are the control of fertilizer F1, 100-108 are the control of F2, 154-162 are the control of F0. Penicillin antibiotic was used once with lower concentration on 54 trees only through injection and spray. The endophyte was used in 14 days after penicillin was injected for penicillin treatments (trees 1-9, 55-63, and 109-117). | | | | | | |  |

**Table S3.** Reduction of *C*Las pathogen concentration (copies/g) using endophyte *Bacillus subtilis* L1-21 and other treatments.

| **Day** | ***Bacillus subtilis* L1-21** | | **Penicillin (100 µg/µl)** | **LB broth** | **Water** |
| --- | --- | --- | --- | --- | --- |
|  | **10^4^** | **10^6^** |  |  |  |
| **0** | 1.12×10^7^ | 1.23×10^6^ | 4.71×10^5^ | 2.38×10^5^ | 1.51×10^6^ |
| **1** | 1.76×10^6^ | 3.58×10^5^ | 2.43×10^5^ | 3.03×10^5^ | 1.59×10^6^ |
| **2** | 9.53×10^5^ | 1.16×10^5^ | 1.51×10^5^ | 2.84×10^5^ | 4.85×10^5^ |
| **3** | 8.92×10^4^ | 6.06×10^4^ | 1.12×10^5^ | 2.49×10^5^ | 3.44×10^6^ |
| **4** | 3.72×10^4^ | 3.93×10^4^ | 1.64×10^4^ | 5.01×10^5^ | 8.20×10^4^ |

*Citrus half leaf method was employed to check the reduction of pathogen in diseased citrus midribs after different treatments. Each treatment comprised three replicates of 12 diseased citrus leaves (6 random top and bottom half leaves each), and the experiment was repeated five times.

**Table S4.** Novel half leaf experiment with different citrus leaves.

| **Days** | ***Bacillus subtilis* L1-21 (10^4^)** | | | ***Bacillus subtilis* L1-21 (10^6^)** | | | **Penicillin (100 µg/µl)** | | | | **LB broth** | | | | **Water** | | |
| --- | --- | --- | --- | --- | --- | --- | --- | --- | --- | --- | --- | --- | --- | --- | --- | --- | --- |
|  | Initial path. | After treat. | **%** redu. | Initial path. | After treat. | **%** redu. | Initial path. | After treat. | **%** redu. | Initial path. | | After treat. | **%** redu. | Initial path. | | After treat. | **%** redu. |
| **0** | 6.72×10^4^ | - | **-** | 1.41×10^5^ | - | **-** | 1.01×10^5^ | - | **-** | 1.34×10^5^ | | - | **-** | 5.17×10^4^ | | - | **-** |
| **1** | 2.67×10^5^ | 1.01×10^5^ | 62.18 | 3.23×10^5^ | 1.68×10^5^ | 48.11 | 9.25×10^5^ | 1.82×10^5^ | 80.32 | 7.85×10^5^ | | 9.70×10^5^ | -23.57 | 1.62×10^5^ | | 5.81×10^5^ | -259.52 |
| **2** | 1.23×10^5^ | 4.38×10^4^ | 64.27 | 1.01×10^5^ | 5.24×10^4^ | 48.00 | 4.16×10^5^ | 1.34×10^5^ | 67.74 | 9.72×10^4^ | | 5.02×10^5^ | -417.12 | 3.58×10^5^ | | 9.71×10^4^ | 72.88 |
| **3** | 2.64×10^5^ | 1.59×10^5^ | 39.64 | 5.06×10^5^ | 7.84×10^4^ | 84.53 | 4.94×10^5^ | 6.51×10^4^ | 86.81 | 5.52×10^5^ | | 2.78×10^5^ | 49.62 | 4.11×10^4^ | | 3.00×10^5^ | -629.80 |
| **4** | 4.05×10^5^ | 3.65×10^3^ | 99.10 | 5.54×10^5^ | 2.29×10^3^ | 99.59 | 4.24×10^5^ | 1.59×10^5^ | 62.39 | 3.36×10^5^ | | 7.99×10^5^ | -137.62 | 9.41×10^4^ | | 2.32×10^5^ | -146.47 |
| **5** | 2.15×10^5^ | 1.22×10^4^ | 94.32 | 3.53×10^5^ | 1.07×10^5^ | 69.76 | 4.57×10^5^ | 4.37×10^4^ | 90.45 | 4.28×10^5^ | | 2.83×10^5^ | 33.86 | 1.93×10^5^ | | 1.63×10^5^ | 15.82 |

*Individual citrus leaves (9 random top and bottom half leaves each in each replicate; 3 replicates in total). This experiment was repeated with different leaves on different days to check the reduction of *C*Las pathogen inside diseased midribs. Percentage reduction was recorded for each replicate on individual day. Experiment was repeated three times. Percentage reduction was calculated by subtracting the number of pathogen after treatment from initial pathogen copies, divided by initial pathogen and multiply by 100.

Note. Path. represent pathogens; treat. represent treatment; and redu. represent reduction; LB, Luria Bertani medium


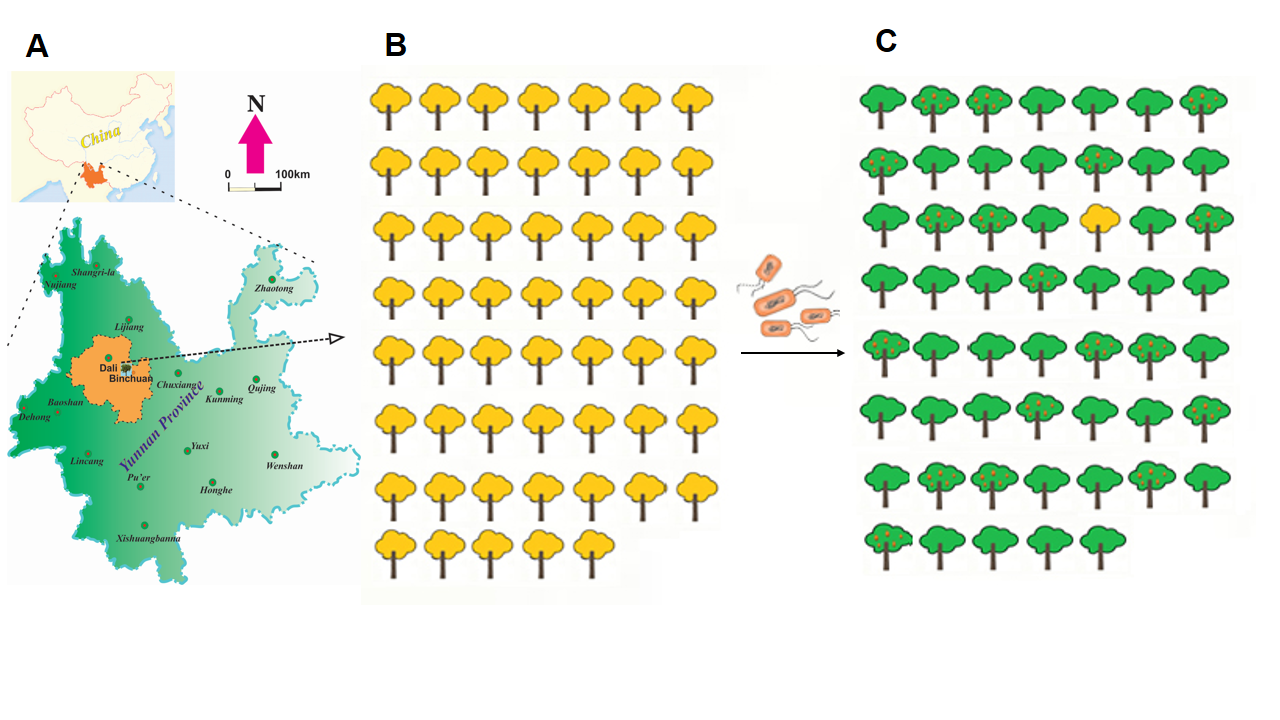


**Fig. S1. Inhibition and exclusion of *C*Las pathogen in diseased fields after application of *B. subtilis* L1-21. (A)** Area with blue colour represents the citrus fields where the experiments were performed in diseased citrus trees; **(B)** diseased citrus fields (yellow colour displayed HLB affected trees) before endophyte *B. subtilis* L1-21 application. A total of 162 diseased citrus trees were treated with the endophyte; **(C)** green trees, and trees with orange fruit depicted the control effect in diseased citrus trees. One citrus tree present in the figure represents a total of three trees in the field. *B. subtilis* L1-21 shown in pink colour represent its characteristics colony morphology.


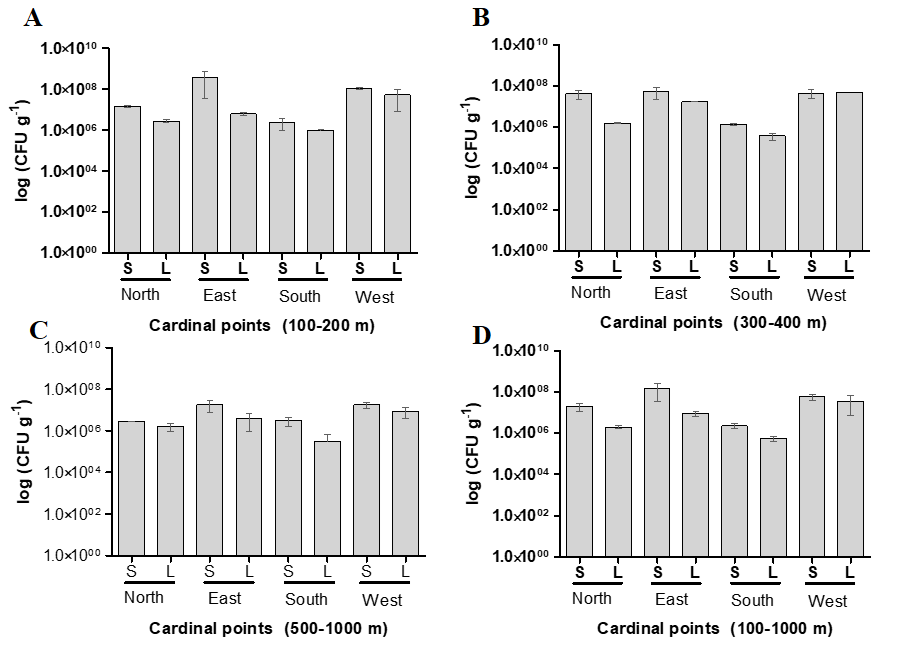


**Fig. S2.** Distribution of the endophyte *Bacillus subtilis* L1-21 across different cardinal points in a citrus grove in Binchuan, Yunnan province, China. Endophyte was sprayed on one specific point in a citrus grove. Leaves samples were collected after 2 weeks from different distances. Each bar represents data of three biological replicates with depicting standard error. Culture-based survey of different leaves including both small (S) and large (L) indicated endophytic cells. Endophyte L1-21 could travel an area/distance range from 100 m to 1000 m across all cardinal points in 2 weeks. Error bars indicate the standard error of the mean (SEM).
